# A 3-month survival model after Gamma Knife surgery in patients with brain metastasis from lung cancer with Karnofsky performance status ≤ 70

**DOI:** 10.1038/s41598-023-40356-6

**Published:** 2023-08-12

**Authors:** Hangeul Park, Hyun-Tai Chung, Jin-Wook Kim, Yun-Sik Dho, Eun Jung Lee

**Affiliations:** 1 Department of Neurosurgery, Seoul National University Hospital, Seoul, Republic of Korea; 2 Gamma Knife Radiosurgery Center, Seoul National University Hospital, Seoul, Republic of Korea; 3 Seoul National University College of Medicine, Seoul, Republic of Korea; 4 Neuro-Oncology Clinic, National Cancer Center, Goyang, Republic of Korea

**Keywords:** CNS cancer, Metastasis, Non-small-cell lung cancer

## Abstract

Gamma Knife surgery (GKS) for brain metastasis (BM) has been generally advocated for patients with a Karnofsky performance status (KPS) scale of ≥ 70. However, some patients with a poor KPS scale of < 70 are recoverable after GKS and show durable survival. A purpose of this study is to devise a 3-month survival prediction model to screen patients with BM with a KPS of ≤ 70 in whom GKS is needed. A retrospective analysis of 67 patients with a KPS scale of 60–70 undergoing GKS for BM of non-small cell lung cancer (NSCLC) from 2016 to 2020 in our institute was performed. Univariate and multivariate logistic regression analyses were performed to investigate factors related to survival for more than 3 months after GKS. The probability (P) prediction model was designed by giving a weight corresponding to the odds ratio of the variables. The overall survival was 9.9 ± 12.7 months (range 0.2–53.2), with a 3-month survival rate of 59.7% (n = 40). In multivariate logistic regression analysis, extracranial disease (ECD) control (p = .033), focal neurological deficit (FND) (p = .014), and cumulative tumor volume (∑ TV) (p = .005) were associated with 3-month survival. The prediction model of 3-month survival (Harrell’s C index = 0.767) was devised based on associated factors. In conclusion, GKS for BMs is recommended in selected patients, even if the KPS scale is ≤ 70.

## Introduction

Stereotactic radiosurgery (SRS) is the most preferred treatment modality for brain metastasis (BM), with a reported local tumor control (LTC) rate of over 80%^1–3^. It has been expandingly applied even for large BMs with the recent introduction of the fractionated SRS system and to multiple BMs over 10, encouraging a more active stance in BM treatment than before^4–6^. Karnofsky performance status (KPS), a well-known prognostic factor for the survival of BM patients, plays a role as an indicator to determine which patients should undergo BM treatment ^7–9^. Recursive partitioning analysis (RPA) and graded prognostic assessment (GPA), which have classified prognosis consistently, demonstrated that KPS < 70 was a poor prognostic factor, with a median survival of 2.3 months in RPA; thus, earnest BM treatment, including SRS, has been generally considered in patients with KPS ≥ 70^10–12^. However, such prognostic systems were introduced 10–20 years ago; meanwhile, the survival of cancer patients has markedly improved owing to advances in chemotherapy and immunotherapy, with a widened application of SRS for BM^13–18^. Therefore, re-establishing the prognostic prediction system for BM patients based on current treatment data is necessary. In this study, we focused on patients with a KPS of 60–70 whose prognosis was generally considered to be borderline, aiming to screen patients with an anticipated life expectancy of more than 3 months and thus who need active SRS for BMs. Considering the confounding effect of the kind of primary tumor, we confined the analyses to BM from non-small cell lung cancer (NSCLC).

## Methods

### Patients

From March 2016 to October 2020, 872 patients underwent Gamma Knife surgery (GKS) for BMs in our institute. Of 113 patients with a KPS of 60–70, 67 patients with 280 BMs from NSCLC were retrospectively analyzed. 46 patients had a primary cancer other than NSCLC and were excluded from the analysis. The data regarding the patient’s demographic features, KPS, focal neurological deficit (FND, present vs. absent), extracranial disease (ECD) status (controlled vs. uncontrolled) at the time of GKS, extracranial metastasis (present vs. absent), gene mutation, concurrent targeted therapy or immunotherapy (TT/IT), and whole-brain radiotherapy (WBRT) before GKS were extracted from electronic medical records. The KPS scale was recorded in all patients. The change in FND after GKS was evaluated one month after GKS. Regarding ECD status, partial response and stable disease were defined as controlled according to RECIST version 1.1 and otherwise as uncontrolled^19^. Concurrent TT/IT was defined as the administration of TT/IT within one month before and after GKS. The effects of concurrent TT/IT timing before and after GKS were separately analyzed. The patients underwent brain MRI at 3-month intervals after GKS to evaluate LTC and intracranial distant failure, with failure of LTC defined as increment tumor volume by > 20% and intracranial distant failure defined as the development of a new lesion. Patient death data were acquired from the National Statistical Office.

### Gamma Knife surgery

All patients underwent GKS using Leksell Gamma Knife ICON (Elekta Instrument AB, Stockholm, Sweden). Treatment planning was performed using Leksell GammaPlan software (Elekta AB). BMs were defined on 1-mm thickness gadolinium-enhanced T1-weighted images, referring to the nonenhanced T1-, T2-weighted images, and black-blood contrast images. Out of the total 67 patients, 45 patients (67.2%) received single-fraction GKS, 18 patients (26.9%) received 3 fractions GKS, and 4 patients (6.0%) received 5 fractions GKS for consecutive days. A thermoplastic mask was generally applied for fractionated GKS, whereas a rigid stereotactic frame was used for single-fraction GKS. Data regarding the number of BMs, intracranial cumulative tumor volume (∑ TV), and prescription dose were extracted from the prospectively collected database. To ensure a fair comparison of the prescription dose among patients with varying numbers of fractions, the doses of patients receiving hypofractionation were converted to an equivalent single fraction dose, considering the biologically effective dose (BED) values. BED was estimated using a linear-quadratic model, assuming an α/β ratio of 10^20^. In patients with multiple BMs, the mean prescription dose and BED were used for comparison.

### Statistical analysis

Statistical analysis was performed using SPSS version 25.0 (IBM Corp., Armonk, NY, USA), and a p value < .05 was considered statistically significant. Survival for more than 3 months after GKS was defined as a favorable outcome and otherwise as a poor outcome. To determine the factors associated with a favorable outcome, multivariate logistic regression analysis was performed with the stepwise variable selection method, including variables with a p value of < .1 in univariate logistic regression. The 3-month survival probability (P) prediction model was designed by giving a weight corresponding to the odds ratio (OR) of the variables identified in the multivariate analysis as follows:

$$\mathrm{P}=\frac{\left({exp}^{\beta 0}\times weight\right)}{1+\left({exp}^{\beta 0}\times weight\right)}$$

To validate the developed prediction model, the c-index was calculated using the leave-one-out cross-validation (LOOCV) approach. Overall survival (OS) was analyzed using the Kaplan‒Meier method and compared using the log-rank test. A minimum p value approach was used with a logistic regression model to estimate the ∑ TV with the greatest difference in OS.

### Ethical statement

Institutional review board of Seoul National University Hospital waived the requirement for informed consent and approved the study protocol and chart review (Approval No. 2101-155-1191). All investigations were conducted in accordance with institutional review board of Seoul National University Hospital guidelines and regulations.

## Results

The OS of the patients was 9.9 ± 12.7 months (range 0.2–53.2), with a 3-month survival rate of 59.7% (n = 40). At the time of GKS, ECD was progressing in 59.7% (n = 40) of patients, with accompanying extracranial metastasis in 64.2% (n = 43). A total of 65.5% of patients (n = 44) had FND due to BMs, of whom 47.7% (n = 21) improved after GKS. The mean number of BMs was 4.2 ± 3.2 (range 1–11), with a ∑ TV of 11.3 ± 12.4 (range 0.2–57.2) cm^3^. The prescription dose of 20.5 ± 1.9 Gy (14–24) was delivered to the tumor, equivalent to a BED_10_ of 63.1 ± 9.1 Gy_10_ (32.7–81.6). Approximately one-third of patients (n = 24, 35.8%) expressed driver mutations, such as EGFR and ALK genes. TT/IT was applied in 62.7% of the patients (n = 42), with 38.8% of patients (n = 26) receiving TT or IT before GKS and 37.3% of patients (n = 25) receiving TT/IT concurrently after GKS. Among the 26 patients who had been receiving TT/IT before GKS, 17 (65%) discontinued the treatment following GKS due to drug resistance without exploring alternative TT/IT options; six switched to different types of TT/IT when diagnosed with BM; and three maintained their medication. The demographic features of the patients are compared between the favorable and poor outcome groups in Table 1. In the favorable group, LTC was achieved 3 months after GKS in 27 patients (81.8%, 27 of 33), and 18 patients (54.5%, 18 of 33) showed intracranial distant failure during the follow-up period. In contrast, most patients in the poor outcome group were not available for evaluation due to premature death before neuroimaging study. Fifty-eight patients (86.6%) died at the time of investigation, and 79% (n = 46) died from uncontrolled ECD.

**Table 1:** Univariate logistic regression of demographic factors associated with survival within three months of Gamma Knife surgery.

Variable	All patients (n = 67)	Favorable outcome (n = 40)	Poor outcome (n = 27)	OR	95% CI	p value
Age at GKS (range)	64.8 ± 11.1 (39–86)	65.1 ± 9.9 (40–81)	64.4 ± 12.9 (39–86)	1.01	0.96–1.05	.814
Male (%)	47 (70.1%)	25 (62.5%)	22 (81.5%)	0.38	0.12–1.21	.102
KPS 60	16 (23.9%)	9 (22.5%)	7 (25.9%)	0.83	0.27–2.59	.387
Disease duration, months (range)	23.3 ± 24.7 (0–108)	28.0 ± 27.3 (0–108)	16.4 ± 18.6 (0–61)	1.02	1.00–1.05	.070
Extracranial disease control	27 (40.3%)	21 (52.5%)	6 (22.2%)	4.08	1.35–12.32	.013
Extracranial metastasis	43 (64.2%)	21 (52.5%)	22 (81.5%)	0.27	0.08–0.84	.025
Focal neurological deficit	46 (68.7%)	34 (85.0%)	12 (44.4%)	5.00	1.69–14.79	.004
Improvement after GKS	21 (45.7%)	18 (52.9%)	3 (25.0%)	1.39	0.51–3.78	.517
Number of lesions (range)	4.2 ± 3.2 (1–11)	3.7 ± 2.3 (1–10)	5.0 ± 3.5 (1–11)	0.88	0.75–1.03	.102
Cumulative tumor volume, cm^3^ (range)	11.3 ± 12.4 (0.2–57.2)	7.9 ± 7.9 (0.31–51.29)	16.4 ± 15.9 (0.2–57.2)	0.94	0.90–0.99	.013
Mean prescription dose, Gy (range)	20.5 ± 1.9 (14–24)	20.6 ± 2.0 (14–24)	20.3 ± 1.7 (16–23)	1.08	0.83–1.40	.569
Mean BED_10_, Gy (range)	63.1 ± 9.1 (32.7–81.6)	63.6 ± 9.7 (32.7–81.6)	62.4 ± 8.2 (43.2–75.9)	1.01	0.96–1.07	.602
EGFR or ALK positive	24 (35.8%)	16 (40%)	8 (29.6%)	1.58	0.56–4.48	.387
TT/IT before GKS	26 (31.3%)	15 (18.5%, n = 39)	11 (40.7%)	0.91	0.33–2.48	.852
TT/IT after GKS	25 (37.3%)	19 (47.5%)	6 (22.2%)	3.50	1.17–10.50	.025
WBRT before GKS	9 (13.4%)	6 (15.0%)	3 (11.1%)	1.41	0.32–6.21	.648
Time to death, months (range)	9.9 ± 12.7 (0.2–53.2)	15.4 ± 14.1 (3.1–53.2)	1.7 ± 0.9 (0.2–2.8)	1.26	1.11–1.44	.001

*ALK* anaplastic lymphoma kinase, *BED* biological equivalent dose, *CI* confidence interval, *EGFR* epidermal growth factor receptor, *GKS* Gamma Knife surgery, *KPS* Karnofsky performance status, *OR* odds ratio, *TT/IT* targeted therapy/immunotherapy, *WBRT* whole-brain radiation therapy.

### Factors associated with 3-month survival after GKS

**Figure 1:**
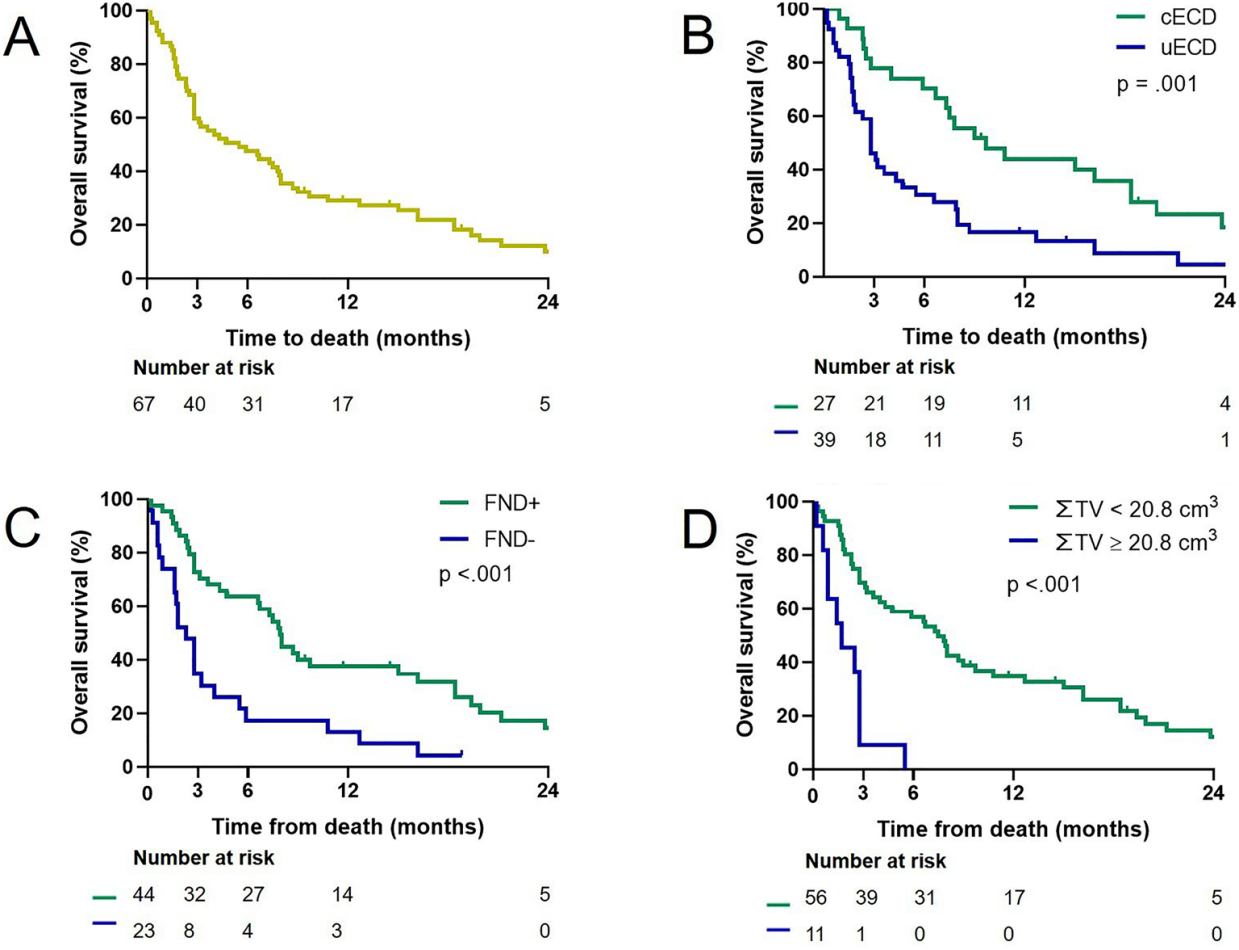
Kaplan‒Meier curves of overall survival. (**A**) The overall survival (OS) of the entire cohort is presented. (**B**) The OS is shown according to the control status of extracranial disease (ECD). cECD; controlled ECD, uECD; uncontrolled ECD. (**C**) The OS according to the presence or absence of focal neurological deficit (FND) at the time of Gamma Knife surgery is presented. + ; present, –; absent. (**D**) The OS according to cumulative tumor volume (∑ TV) is presented. The cut-off value of 20.8 cm3, which maximizes the difference in OS between the two groups, was used to dichotomize the groups.

In univariate logistic regression analysis, ECD control (p = .013), absence of extracranial metastasis (p = .025), presence of FND at the time of GKS (p = .004), concurrent post-GKS TT/IT (p = .025), and smaller ∑ TV (p = .013) were significantly associated with a favorable outcome (Table 1). In multivariate logistic regression analysis, the factors associated with a favorable outcome were controlled ECD (OR 4.13, 95% CI 1.13–15.15, p = .033), presence of FND (OR 4.98, 95% CI 1.39–17.87, p = .014), and ∑ TV (OR per cm^3^ 0.92, 95% CI 0.87–0.98, p = .005) (Table 2). The ∑ TV cut-off value with the largest OS difference between the two groups was 20.8 cm^3^ via the minimum p value approach (OR 3.24, 95% CI 1.55–6.74, p = .002). The Kaplan‒Meier curves of OS for each prognostic factor of a favorable outcome are presented in Fig. 1.

**Table 2:** Coefficient (b) of each parameter as estimated using multivariate logistic regression and its estimated weight.

Variable	b(SE)	p value	OR	95% CI	Weight
Intercept	− 0.2505(0.5411)	.643			
Extracranial disease control					
Controlled	1.4182(0.6633)	.033	4.13	1.13–15.15	4
Uncontrolled	0		0		0
Focal neurological deficit					
Present	1.6054(0.6519)	.014	4.98	1.39–17.87	5
Absent	0		0		0
Cumulative tumor volume (cm^3^)	− 0.083(0.0296)	.005	0.92	0.87–0.98	0.9

*CI* confidence interval, *OR* odds ratio, *SE* standard error.

### A predictive model for 3-month survival after GKS

ECD, FND, and ∑ TV were included as parameters to develop a predictive model for the 3-month survival of NSCLC patients with poor KPS undergoing GKS for BM. ECD was categorized into controlled and uncontrolled groups, and FND was categorized into present and absent groups. In contrast, ∑ TV was applied as a continuous variable with a unit of cm^3^. The impact of each parameter on survival was weighed based on their OR values in multivariate analysis. The weights of ECD and FND were determined by converting the OR values to the nearest integer, while that of ∑ TV was determined by rounding the OR to two decimal places to avoid the weight being 1 (Table 2). Consequently, the probability (P) of 3-month survival was estimated using the following equation:

1 $$\mathrm{P}=\frac{\left({exp}^{\beta 0}\times weight\right)}{1+\left({exp}^{\beta 0}\times weight\right)}= \frac{\left({exp}^{-0.2505}\times \left({4}^{a}\times {5}^{b}\times {0.9}^{c}\right)\right)}{1+\left({exp}^{-0.2505}\times \left({4}^{a}\times {5}^{b }\times {0.9}^{c}\right)\right)}$$

where a corresponds to ECD, with controlled = 1 and uncontrolled = 0; b corresponds to FND, with present = 1 and absent = 0; and c indicates ∑ TV. Harrell’s C index was 0.767 (95% CI 0.642–0.891). 3-month survival calculator is included in Supplement 1. Four categories are generated by combining the ECD and FND conditions: (1) controlled ECD with FND (n = 25); (2) uncontrolled ECD with FND (n = 21); (3) controlled ECD without FND (n = 3); and 4) uncontrolled ECD without FND (n = 18). For the convenience of screening patients with a likelihood of 3-month survival, we aimed to suggest the cut-off ∑ TV (c) in each categorical group, and the c corresponding to each P was calculated using Eq. 2 converted from Eq. 1 as follows.

2 $$\mathrm{ln}\frac{P}{1-P}=-0.2505+ln4\times a+ln5\times b+ln0.9\times c$$

**Figure 2:**
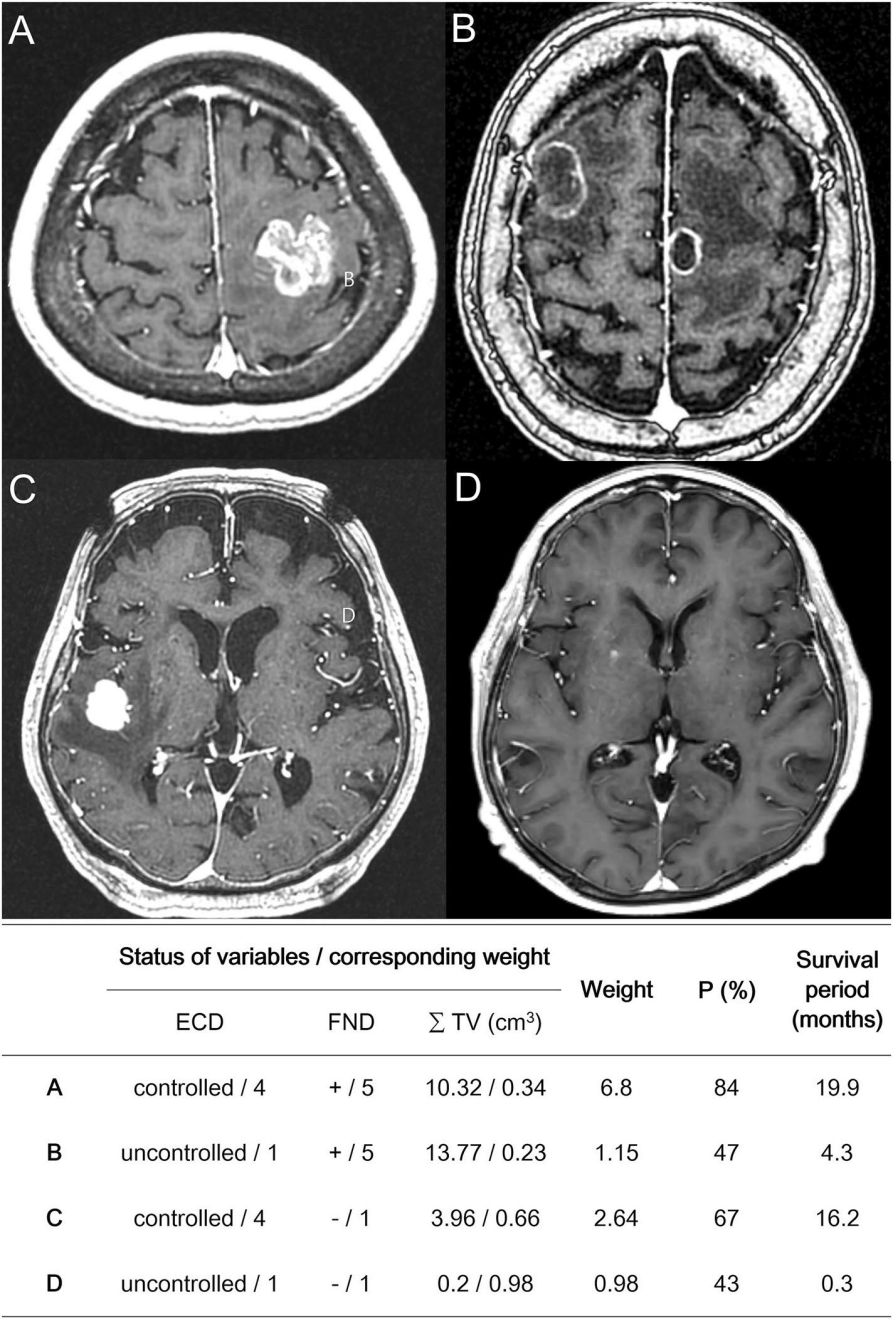
The application of a predictive model for 3-month survival after Gamma Knife surgery (GKS) is presented. (**A**–**D**) represent the cases belonging to categories 1, 2, 3, and 4, respectively. The probabilities (P) of 3-month survival after GKS of each case were calculated using Eq. 1, which showed a good agreement with the actual survival period in broad outlines. ECD; extracranial disease, FND; focal neurological deficit, ∑ TV; cumulative tumor volume, + ; present, –; absent, P (%); the probability of 3-month survival.

**Figure 3:**
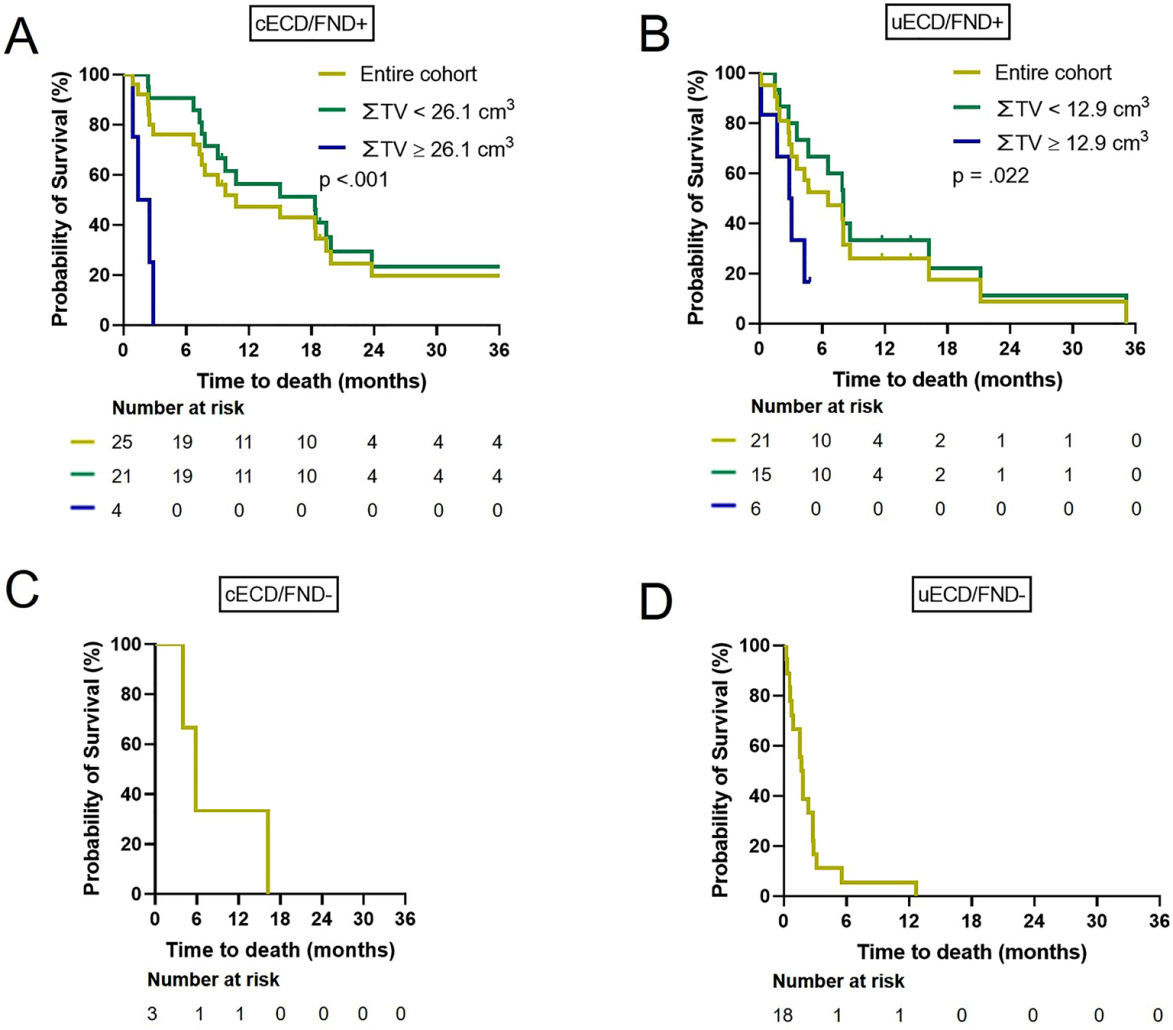
Kaplan‒Meier curves of overall survival (OS) in each category. (**A**), (**B**), (**C**), and (**D**) indicate categories 1,2,3, and 4, respectively. (**A**) and (**B**) show OS according to the cumulative tumor volume (∑ TV) with a corresponding cut-off value of each category for a 50% probability of survival ≥ 3 months after Gamma Knife surgery. Most patients in category 1 survive more than 3 months unless their ∑ TV is enormous, and thus active treatment is appropriate. In contrast, most patients in category 4 show premature death, and supportive care is suitable. cECD; controlled extracranial disease, FND; focal neurological deficit, uECD; uncontrolled ECD, +; present, –; absent.

The results are summarized in Table 3. For example, when we screened patients with a 3-month survival probability of ≥ 50%, the maximum acceptable ∑ TV values for GKS were 26.1 cm^3^, 12.9 cm^3^, and 10.8 cm^3^ in categories 1, 2, and 3, respectively. However, all patients in category 4 showed a 3-month survival probability of less than 50%, with 83.3% of patients (15 of 18) dying within 3 months. Figure 2 shows a representative case of each category to demonstrate how this predictive model is applied. The Kaplan‒Meier curves for OS in categories 1, 2, 3, and 4 are presented in Fig. 3.

**Table 3:** The cut-off of cumulative tumor volume for each 3-month survival probability (P).

P	Cut-off of cumulative tumor volume (cm^3^)			
	Category 1	Category 2	Category 3	Category 4
0.1	46.9	33.8	31.6	18.5
0.2	39.2	26.1	23.9	10.8
0.3	34.1	20.9	18.8	5.7
0.4	29.9	16.7	14.6	1.5
0.5	26.1	12.9	10.8	− 2.4
0.6	22.2	9.0	6.9	− 6.2
0.7	18.0	4.9	2.7	− 10.4
0.8	12.9	− 0.3	− 2.4	− 15.5
0.9	5.2	− 8.0	− 10.1	− 23.2

Category 1, controlled ECD and presence of FND; Category 2, uncontrolled ECD and presence of FND; Category 3, controlled ECD and absence of FND; Category 4, uncontrolled ECD and absence of FND; ECD, extracranial disease; FND, focal neurological deficit.

## Discussion

### The justification of a new prognostic system for BM patients with low KPS scores

SRS is an established treatment for BM and is preferred over open surgery and WBRT^21^. With the recent introduction of the fractionated SRS system, SRS application has been extended to large tumors that previously required open surgery^22,23^. Additionally, SRS allows concurrent TT/IT, unlike open surgery, facilitating the treatment of systemic disease while minimizing the interruption of chemotherapy; this may have contributed to improvement in the survival of BM patients along with the advancement of chemotherapy^24,25^. Moreover, it has been demonstrated that even multiple BMs over 10 can be effectively treated with SRS, which is advantageous for conserving cognitive function compared to WBRT ^4,21^. Active treatment for BM used to be recommended in patients with KPS ≥ 70^4^; however, advances in chemotherapy and the expansion of SRS application have increased the treatment options for BM patients with KPS < 70, which may further intensify the dependence on SRS for BM treatment^26,27^. Therefore, it is essential to identify the appropriate candidates for SRS among BM patients with KPS < 70. This consideration is vital to ensure the adequate allocation of limited medical resources to those with a higher likelihood of prolonged survival, while also enabling patients in a dire state to prepare for their end-of-life rather than enduring ineffective treatments.

### *A novel 3-month survival probability model in patients with a KPS score of* ≤ *70*

**Figure 4:**
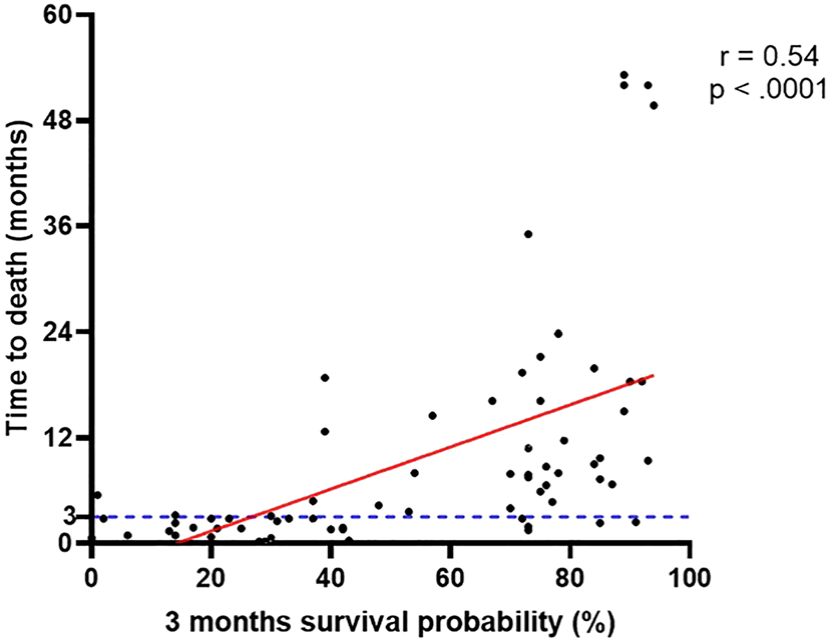
The correlation between the estimated 3-month survival probability derived from the model and the actual survival after Gamma Knife surgery.

In this study, we defined patients with a life expectancy of over 3 months as needing active treatment. We investigated the factors associated with 3-month survival in patients with a KPS of 60–70, generally representing poor and borderline prognosis by dichotomous classification, who underwent GKS for BMs from NSCLC. As a result, controlled ECD, the presence of FND, and small ∑ TV were significantly associated with a survival time of over 3 months after GKS for BM. These results are consistent with previously published literature. ECD is a well-known prognostic factor of BM patients, and uncontrolled ECD is the leading cause of death in BM, as shown in this study^28,29^. KPS generally reflects the ECD status, and those with a KPS that worsens with ECD progression do not seem to recover well. This may be why the KPS has been a robust prognostic factor in cancer patients in many studies to date. However, patients presenting FND due to BM have a poor KPS scale but can recover after SRS for BMs. Notably, this study was confined to patients with a KPS of 60–70, and the presence of FND was given a higher weight than ECD for 3-month survival as 5 versus 4, which had the most substantial impact on survival among the three demonstrated factors until the ∑ TV was < 15.3 cm^3^. Similar to our study, Chernov and colleagues studied the prognostic factors of patients with KPS ≤ 50 who underwent GKS for BM and demonstrated that low KPS due to BM-derived FND was associated with favorable survival, while low KPS resulting from ECD was related to a poor prognosis^26^. Several studies have demonstrated ∑ TV as another important prognostic factor; the larger ∑ TV is, the more negative the influence on survival^2,30–32^. This study showed that the effect of ∑ TV was the greatest among the three prognostic factors when ∑ TV was ≥ 15.3 cm^3^. At first, these results seem to conflict with the finding that most BM patients die from uncontrolled ECD, not from BM itself^4,33,34^. Based on this, we speculate that ∑ TV reflects the severity and progression rate of the disease independently of KPS and is, therefore, closely related to OS. We categorized the patients into four groups based on their ECD and FND statuses and calculated the cut-off ∑ TV corresponding to the 3-month survival probability ranging from 10 to 90% based on the statistical model (Table 3). According to the model, while a large ∑ TV is acceptable for SRS in category 1, a smaller ∑ TV is treatable in categories 2 and 3, and in category 4, a poor prognosis is predicted in most cases. Therefore, we suggest active SRS for patients in category 1 unless their ∑ TV is enormous, whereas supportive care for patients in category 4. Although the 3-month survival probability model was developed based on dichotomized survival data, the result value is continuous data calculated in the context of the weight of each variable. Also, a notable correlation was demonstrated that a higher 3-month survival probability was linked to a more prolonged time to death (Pearson correlation r = 0.54, p < .0001) (Fig. 4). Hence, the suggested 3-month survival probability model effectively captures the actual life expectancy, even considering the limited sample size, and we believe this model can assist clinicians in determining whether the patient is amenable to GKS.

### Factors associated the survival in NSCLC patients with BM in the literature

It has been reported that EGFR mutations and ALK rearrangements play a significant role in the invasion of circulating tumor cells through the blood–brain barrier (BBB) and tumor angiogenesis, resulting in BM in NSCLC patients^35,36^. Therefore, targeting these genetic abnormalities with drugs capable of penetrating the BBB has shown efficacy in treating BM of NSCLC, and some studies have reported a potential reduction in the occurrence of BM^37^. Recent studies have reported that EGFR tyrosine kinase inhibitors improved the median OS to > 16 months and ALK inhibitors significantly improved the median OS to > 40 months compared to 7.2–7.8 months of the conventional chemotherapy era^38–42^. This study demonstrated that the concurrent administration of TT/IT after GKS was significantly associated with OS in the Kaplan–Meier analysis (Supplement 2). On univariate logistic regression analysis with the endpoint of the 3-month survival, the concurrent use of TT/IT following GKS still had a significant association, while it was not determined as an independent factor on multivariate analysis. We conducted an additional analysis to ascertain the genuine impact of TT/IT by excluding three patients who continued the same treatment they had been receiving prior to the development of BM, considering the possible drug resistance. On multivariable Cox proportional regression analysis, concurrent TT/IT following GKS demonstrated significant improvement in OS (HR 2.030, 95% CI 1.087–3.789, p = .026), in line with previous studies. However, in logistic regression analysis with the endpoint of 3-month survival, concurrent TT/IT did not show a correlation on multivariable analysis, while it was significantly associated with 3-month survival on univariable analysis (HR 5.202, 95% CI 1.521–17.796, p = .009). The limited statistical power of TT/IT in the multivariate logistic analysis could be attributed to the small sample size. Otherwise, the impact of poor disease status, characterized by uncontrolled ECD and substantial ∑ TV, on premature mortality within 3 months might outweigh the potential advantages of TT/IT. Meanwhile, the use of TT/IT before GKS did not show any association with OS or 3-month survival. Among these patients, most patients (n = 20, 77%) were considered to develop drug resistance. There was no significant difference in 3-month survival between patients who demonstrated resistance to targeted TT/IT and those who did not. Given the limited sample size and the complexities of the individual treatment course, compounded by the various systemic chemotherapy regimens, this study could not conclude the significance of disease refractoriness to TT/IT on early mortality after GKS. In addition, age and number of BMs, which were included as prognostic factors in other predictive systems, such as RPA, GPA, and the score index for radiosurgery, were not associated with 3-month survival in this study^12,43,44^. We consider that the inconclusive results on other strong prognostic factors in this study might have been attributed to the small sample size and the narrow KPS range of the patients. Nevertheless, this study, which focused on patients with KPS ≤ 70, known as borderline-to-poor prognosis, provides insight into screening patients who are suitable to undergo active SRS despite the low KPS score by suggesting a statistical prediction model for 3-month survival.

### Limitations

This research was based on a retrospective single-center study with a relatively small sample size. Our hospital is among the leading tertiary referral hospital in South Korea, where a substantial number of cancer patients receive treatment, and the demand for GKS for BM at our hospital is exceptionally high, with approximately 300 patients undergoing GKS for BM per year. However, this study specifically focused on patients with NSCLC and poor KPS of ≤ 70. In addition, to mitigate any potential confounding influences stemming from improved survival with advances in chemotherapy over time, we recruited patients who were treated contemporaneously, excluding those who had undergone GKS more than 5 years prior to the study design. As a result, only 67 patients were finally included despite a high volume of BM patients receiving GKS. In addition, the proposed prediction model for 3-month survival has a limitation in terms of generalizability, as it has not undergone external validation. However, we believe this study can serve as a stepping stone to future prospective large-scale multicenter studies to develop a more robust survival prediction model for patients with BM.

## Conclusions

Despite having a poor KPS, certain patients with controlled ECD, BM-caused low KPS, and a small intracranial tumor burden exhibit durable survival after GKS. Our predictive model for 3-month survival will assist clinicians in identifying suitable candidates for GKS.

### Supplementary Information

Supplementary Information 1.

Supplementary Information 2.

## Data Availability

The datasets generated during and/or analyzed during the current study are available from the corresponding author on reasonable request.

## References

[1] Bowden G (2015). Gamma knife radiosurgery for the management of cerebral metastases from non-small cell lung cancer. J. Neurosurg..

[2] Ito D (2020). Comparison of two-stage Gamma Knife radiosurgery outcomes for large brain metastases among primary cancers. J. Neurooncol..

[3] Jeong WJ (2015). Efficacy and safety of fractionated stereotactic radiosurgery for large brain metastases. J. Korean Neurosurg. Soc..

[4] Yamamoto M (2014). Stereotactic radiosurgery for patients with multiple brain metastases: A case-matched study comparing treatment results for patients with 2–9 versus 10 or more tumors. J. Neurosurg..

[5] Salvetti DJ, Nagaraja TG, McNeill IT, Xu Z, Sheehan J (2013). Gamma Knife surgery for the treatment of 5 to 15 metastases to the brain: Clinical article. J. Neurosurg..

[6] Graber JJ, Cobbs CS, Olson JJ (2019). Congress of neurological surgeons systematic review and evidence-based guidelines on the use of stereotactic radiosurgery in the treatment of adults with metastatic brain tumors. Neurosurgery.

[7] Sperduto PW (2020). Survival in patients with brain metastases: Summary report on the updated diagnosis-specific graded prognostic assessment and definition of the eligibility quotient. J. Clin. Oncol..

[8] Venur VA, Ahluwalia MS (2015). Prognostic scores for brain metastasis patients: Use in clinical practice and trial design. Chin. Clin. Oncol..

[9] Lee CC, Yen CP, Xu Z, Schlesinger D, Sheehan J (2014). Large intracranial metastatic tumors treated by Gamma Knife surgery: Outcomes and prognostic factors. J. Neurosurg..

[10] Yamamoto M (2014). Stereotactic radiosurgery for patients with multiple brain metastases (JLGK0901): A multi-institutional prospective observational study. Lancet Oncol..

[11] Sperduto PW (2010). Diagnosis-specific prognostic factors, indexes, and treatment outcomes for patients with newly diagnosed brain metastases: A multi-institutional analysis of 4,259 patients. Int. J. Radiat. Oncol. Biol. Phys..

[12] Gaspar L (1997). Recursive partitioning analysis (RPA) of prognostic factors in three Radiation Therapy Oncology Group (RTOG) brain metastases trials. Int. J. Radiat. Oncol. Biol. Phys..

[13] Aguilar A (2022). Impact of targeted therapy on the survival of patients with advanced-stage non-small cell lung cancer in Oncosalud-AUNA. Cancer Control.

[14] Niranjan A, Monaco E, Flickinger J, Lunsford LD (2019). Guidelines for multiple brain metastases radiosurgery. Prog. Neurol. Surg..

[15] Kim KH (2019). Outcome evaluation of patients treated with fractionated Gamma Knife radiosurgery for large (> 3 cm) brain metastases: A dose-escalation study. J. Neurosurg..

[16] Stirrups R (2018). Osimertinib improves progression-free survival in NSCLC. Lancet Oncol..

[17] Di Lorenzo R, Ahluwalia MS (2017). Targeted therapy of brain metastases: Latest evidence and clinical implications. Ther. Adv. Med. Oncol..

[18] Shaw AT (2020). First-line lorlatinib or crizotinib in advanced ALK-positive lung cancer. N. Engl. J. Med..

[19] Eisenhauer EA (2009). New response evaluation criteria in solid tumours: Revised RECIST guideline (version 1.1). Eur. J. Cancer.

[20] Fowler JF (2010). 21 years of biologically effective dose. Br. J. Radiol..

[21] O'Beirn M (2018). The expanding role of radiosurgery for brain metastases. Medicines.

[22] Kim JW (2016). Fractionated stereotactic gamma knife radiosurgery for large brain metastases: A retrospective, single center study. PLoS ONE.

[23] Park HR (2019). Frameless fractionated Gamma Knife radiosurgery with ICON for large metastatic brain tumors. J. Korean Med. Sci..

[24] McDermott MW, Sneed PK (2005). Radiosurgery in metastatic brain cancer. Neurosurgery.

[25] Sheehan JP, Sun MH, Kondziolka D, Flickinger J, Lunsford LD (2002). Radiosurgery for non-small cell lung carcinoma metastatic to the brain: Long-term outcomes and prognostic factors influencing patient survival time and local tumor control. J. Neurosurg..

[26] Chernov MF (2007). Outcome after radiosurgery for brain metastases in patients with low Karnofsky performance scale (KPS) scores. Int. J. Radiat. Oncol. Biol. Phys..

[27] Yomo S, Hayashi M (2014). A minimally invasive treatment option for large metastatic brain tumors: Long-term results of two-session Gamma Knife stereotactic radiosurgery. Radiat. Oncol..

[28] Gao HX (2018). Comparison of prognostic indices in NSCLC patients with brain metastases after radiosurgery. Int. J. Biol. Sci..

[29] Park JY (2015). Gamma knife radiosurgery for elderly patients with brain metastases: Evaluation of scoring systems that predict survival. BMC Cancer.

[30] Likhacheva A (2013). Predictors of survival in contemporary practice after initial radiosurgery for brain metastases. Int. J. Radiat. Oncol. Biol. Phys..

[31] Baschnagel AM (2013). Tumor volume as a predictor of survival and local control in patients with brain metastases treated with Gamma Knife surgery. J. Neurosurg..

[32] Donofrio CA (2020). Cumulative intracranial tumour volume prognostic assessment: A new predicting score index for patients with brain metastases treated by stereotactic radiosurgery. Clin. Exp. Metastasis.

[33] Liu Y (2015). Salvage whole brain radiotherapy or stereotactic radiosurgery after initial stereotactic radiosurgery for 1–4 brain metastases. J. Neurooncol..

[34] Gondi V (2014). Preservation of memory with conformal avoidance of the hippocampal neural stem-cell compartment during whole-brain radiotherapy for brain metastases (RTOG 0933): A phase II multi-institutional trial. J. Clin. Oncol..

[35] Breindel JL (2013). EGF receptor activates MET through MAPK to enhance non-small cell lung carcinoma invasion and brain metastasis. Cancer Res..

[36] Hida T (2017). Alectinib versus crizotinib in patients with ALK-positive non-small-cell lung cancer (J-ALEX): An open-label, randomised phase 3 trial. Lancet.

[37] Erickson AW, Das S (2019). The impact of targeted therapy on intracranial metastatic disease incidence and survival. Front. Oncol..

[38] Camidge DR (2021). Brigatinib versus Crizotinib in ALK inhibitor-naive advanced ALK-positive NSCLC: Final results of phase 3 ALTA-1L trial. J. Thorac. Oncol..

[39] Reungwetwattana T (2018). CNS response to osimertinib versus standard epidermal growth factor receptor tyrosine kinase inhibitors in patients with untreated EGFR-mutated advanced non-small-cell lung cancer. J. Clin. Oncol..

[40] Moro-Sibilot D (2015). Non-small cell lung cancer patients with brain metastases treated with first-line platinum-doublet chemotherapy: Analysis from the European FRAME study. Lung Cancer.

[41] Ali A, Goffin JR, Arnold A, Ellis PM (2013). Survival of patients with non-small-cell lung cancer after a diagnosis of brain metastases. Curr. Oncol..

[42] Xie L (2019). Osimertinib for EGFR-mutant lung cancer with brain metastases: Results from a single-center retrospective study. Oncologist.

[43] Weltman E (2000). Radiosurgery for brain metastases: A score index for predicting prognosis. Int. J. Radiat. Oncol. Biol. Phys..

[44] Sperduto PW (2012). Summary report on the graded prognostic assessment: An accurate and facile diagnosis-specific tool to estimate survival for patients with brain metastases. J. Clin. Oncol..

