# Supplementary material for: A 3-month survival model after Gamma Knife surgery in patients with brain metastasis from lung cancer with Karnofsky performance status ≤ 70
Source: Sci Rep. 2023 Aug 12;13:13159. doi: 10.1038/s41598-023-40356-6 (PMC10423256; doi:10.1038/s41598-023-40356-6)
Supplement: Supplementary file 2 — Supplementary Information 2. [file 41598_2023_40356_MOESM2_ESM.pptx]

## Slide 1
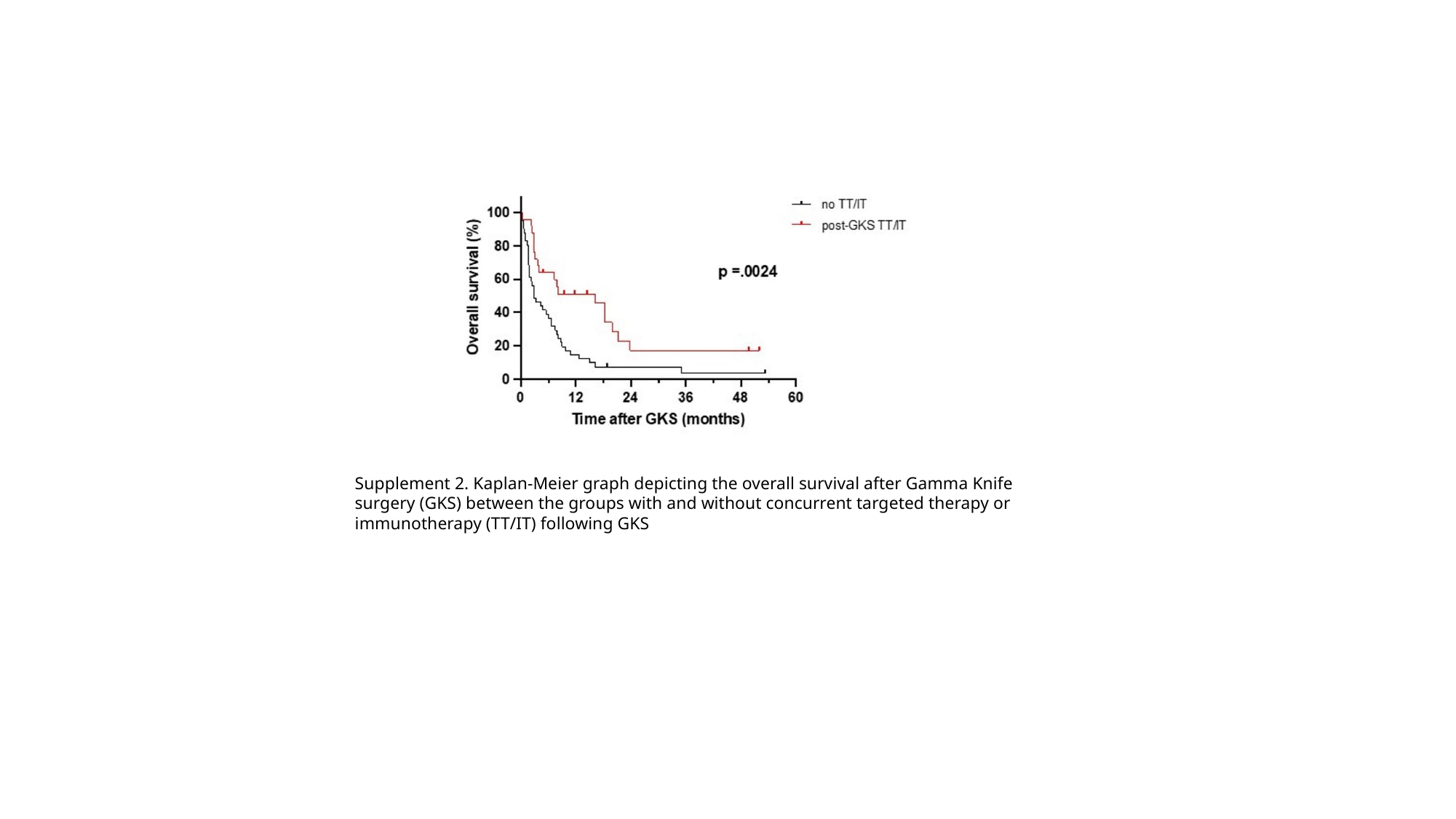

Supplement 2. Kaplan-Meier graph depicting the overall survival after Gamma Knife surgery (GKS) between the groups with and without concurrent targeted therapy or immunotherapy (TT/IT) following GKS
